# Soluble receptors for advanced glycation end products and receptor activator of NF-κB ligand serum levels as markers of premature labor

**DOI:** 10.1186/s12884-015-0559-3

**Published:** 2015-06-10

**Authors:** Rafał Rzepka, Barbara Dołęgowska, Daria Sałata, Aleksandra Rajewska, Marta Budkowska, Leszek Domański, Sebastian Kwiatkowski, Wioletta Mikołajek-Bedner, Andrzej Torbé

**Affiliations:** Department of Obstetrics and Gynecology, Pomeranian Medical University, Al. Powstańców Wielkopolskich 72, 70-111 Szczecin, Poland; Department of Laboratory Diagnostics and Molecular Medicine, Pomeranian Medical University, Al. Powstańców Wielkopolskich 72, 70-111 Szczecin, Poland; Department of Nephrology, Transplantology and Internal Medicine, Pomeranian Medical University, Al. Powstańców Wielkopolskich 72, 70-111 Szczecin, Poland

**Keywords:** Preterm labor, Soluble receptors for advanced glycation end products, sRANKL, Chronic inflammation, pPROM

## Abstract

**Background:**

This study aimed to determine the relationships between secretory and endogenous secretory receptors for advanced glycation end products (sRAGE, esRAGE), sRANKL, osteoprotegerin and the interval from diagnosis of threatened premature labor or premature rupture of the fetal membranes to delivery, and to evaluate the prognostic values of the assessed parameters for preterm birth.

**Methods:**

Ninety women between 22 and 36 weeks’ gestation were included and divided into two groups: group A comprised 41 women at 22 to 36 weeks’ gestation who were suffering from threatened premature labor; and group B comprised 49 women at 22 to 36 weeks’ gestation with preterm premature rupture of the membranes. Levels of sRAGE, esRAGE, sRANKL, and osteoprotegerin were measured. The Mann–Whitney test was used to assess differences in parameters between the groups. For statistical analysis of relationships, correlation coefficients were estimated using Spearman’s test. Receiver operating characteristics were used to determine the cut-off point and predictive values.

**Results:**

In group A, sRAGE and sRANKL levels were correlated with the latent time from symptoms until delivery (r = 0.422; r = −0.341, respectively). The sensitivities of sRANKL and sRAGE levels for predicting preterm delivery were 0.895 and 0.929 with a negative predictive value (NPV) of 0.857 and 0.929, respectively. In group B, sRAGE and sRANKL levels were correlated with the latent time from pPROM until delivery (r = 0.381; r = −0.439). The sensitivity of sRANKL and sRAGE for predicting delivery within 24 h after pPROM was 0.682 and 0.318, with NPVs of 0.741 and 0.625, respectively. Levels of esRAGE and sRANKL were lower in group A than in group B (median = 490.2 vs 541.1 pg/mL; median = 6425.0 vs 11362.5 pg/mL, respectively).

**Conclusions:**

Correlations between sRAGE, sRANKL, and pregnancy duration after the onset of symptoms suggest their role in preterm delivery. The high prognostic values of these biomarkers indicate their usefulness in diagnosis of pregnancies with threatened premature labor.

## Background

Preterm birth remains one of the most important causes of neonatal morbidity and mortality, despite recent considerable development of perinatal medicine [1]. There are many risk factors of premature delivery, including infections, poor socioeconomic status, demographic conditions, as well as environmental and genetic effects [2–6].

Markers are required to not only classify a pregnant woman as being at risk of preterm delivery, but also for implementing adequate and effective prophylaxis. Research conducted in recent years has particularly focused on the role of markers of acute inflammation in etiology and diagnosis of premature labor [7–11].

Osteoprotegerin (OPG) is a glycoprotein that belongs to the family of tumor necrosis factor (TNF) receptors [12]. OPG is produced in endothelial cells, vascular smooth muscle cells, and different cells of the immune system [13–17]. Some proinflammatory cytokines can increase this process, while glucocorticosteroids, parathormone, and fibroblast growth factor decrease this process [18, 19].

Receptor activator of NF-κB ligand (RANKL) is a type II cell membrane glycoprotein from the family of TNF proteins [20]. In the human system, RANKL is present in three forms as a cytoplasmic molecule, as an originally membrane-bound particle, and as a free plasmatic fraction, so-called soluble RANKL (sRANKL) [21, 22]. RANKL is present in osteoblasts, T-lymphocytes, within peripheral lymph nodes, bones, and the fetal liver [20]. Expression of the RANKL gene increases under the action of interleukin-1β, interleukin-11, TNF-α, prostaglandin E2, lipopolysaccharide D3 vitamin D3, and parathormone [23–25].

Receptor activator of nuclear factor kappa-B (NF-κB) (RANK) also belongs to the TNF family [26, 27]. RANK acts as a receptor for RANKL and OPG. After binding of RANK (as a receptor) with RANKL (as its ligand), the receptor undergoes trimerization, which initiates an intracellular cascade, leading to cellular activation [26, 28].

The OPG/RANKL/RANK system plays an important role in bone tissue function, but the reciprocal relation between OPG/RANKL/RANK and the immune system suggests that activation of the immune system in preterm labor can also noticeably affect the OPG/RANKL/RANK system [29, 30]. Even low levels of cytokines can influence components of the OPG/RANKL/RANK system. However, there is a paucity of scientific data to support this hypothesis.

Receptors for advanced glycation end products (RAGE) are nonspecific multiligand receptors that belong to the superfamily of immunoglobulin. Activation of RAGE induces and supports inflammatory responses, mainly by NF-κB and mitogen-activated protein kinase (MAPK) activation. [31–33].

In contrast to native RAGE, negative isoforms have also been described, including secretory RAGE (sRAGE) and endogenous secretory RAGE (esRAGE) [34]. Binding of advanced glycation end products (AGE) and some alarmines to negative RAGE fulfills an important role, preventing the toxic influence of ligand-RAGE complexes [34, 35].

The hypothesis of the protective role of RAGE negative variants and its ligands leads to the question of whether soluble RAGE levels in pregnancy can affect the prevalence of premature labor associated with spontaneous uterine contractility and preterm rupture of the membranes. Only a few authors have investigated RAGE in premature labor [36–42].

We therefore investigated the following. (1) The relationships between levels of sRANKL, OPG, sRAGE, and esRAGE and the interval from the diagnosis of threatened premature labor or preterm premature rupture of the fetal membranes (pPROM) to delivery and evaluated the prognostic value of parameters for preterm birth. (2) The relationships between sRANKL, OPG, sRAGE, and esRAGE levels and other parameters used in diagnosing premature labor. (3) Plasma sRANKL, OPG, sRAGE, and esRAGE levels in pregnancies complicated by threatened premature labor with and without premature rupture of the membranes.

## Methods

This study was conducted in the Department of Obstetrics and Gynecology and in the Department of Laboratory Diagnostics and Molecular Medicine of Pomeranian Medical University from October 29, 2012 to July 30, 2014. The study was approved by the Bioethical Committee of Pomeranian Medical University (KB-0012/121/12). All women gave their written informed consent prior to their inclusion in the study. Ninety women who were between 22 and 36 weeks of gestation were included and divided into two groups. Group A comprised 41 women between 22 and 36 weeks of gestation, presenting with symptoms of threatened premature labor. Group B comprised 49 women between 22 and 36 weeks of gestation with preterm premature rupture of the membranes. The detailed characteristics of the study groups are shown in Table 1. Successive patients who reported to the departments and met the criteria for inclusion were included in the study. Random selection was the method of inclusion.

**Table 1:** General characteristics of the study population

Parameter	Group A	Group B	p value
Number of women	41	49	-
Age (years)	28.32 ± 6.44	30 ± 6.50	NS
Gestational age (weeks)	30.9 ± 3.1	31.10 ± 3.76	NS
Parity	2 ± 1	2 ± 1	NS
Gestational age at delivery (weeks)	34.87 ± 4.04	31.67 ± 3.74	0,001
Birth weight (g)	2547.48 ± 833.41	1939.37 ± 801.77	0,001
Smoker (N)	2	6	NS
Non-smoker (N)	39	43	
Previous history of preterm birth (N)	4	6	NS
No preterm birth history (N)	37	43	
Place of residence – city (N)	31	35	NS
Place of residence – village (N)	10	14	
Excellent socioeconomic status (N)	14	19	NS
Mediocre socioeconomic status (N)	27	30	
Positive cervical culture (N)	14	20	NS
Negative cervical culture (N)	27	29	

Values are mean ± standard deviation (analyzed by Student’s *t*-test) or N (analyzed by *χ* ^2^ Pearson’s test)

The criteria of inclusion in group A were as follows: (1) the presence of spontaneous uterine contractility between 22 and 36 weeks of gestation, with a frequency of at least four contractions per hour within at least a 2-h period, as confirmed in a tocodynamometric test; (2) cervical effacement, as shown in an ultrasound scan, with cervix length < 25 mm; and (3) cervical maturation with a Bishop score ≥ 4. The criteria for inclusion in group B were as follows:(1) diagnosis of premature rupture of the membranes between 22 and 36 weeks of gestation; (2) confirmation of premature rupture of the membranes by a positive test result for the presence of insulin-like growth factor binding protein-1 in vaginal discharge; and (3) absence of preterm spontaneous uterine contractility with a negative tocodynamometric test result.

No later than 2 h after admission to the departments, peripheral maternal blood was sampled from the ulnar vein and put into tubes containing EDTA-K2. After centrifugation (10 min, 5000 rps), plasma samples were stored at −80 °C until measurement of sRAGE, esRAGE, sRANKL, and OPG levels.

Immunoassay methods were used to measure sRAGE, esRAGE, sRANKL,and OPG levels. Human sRAGE ELISA (Bio Vendor Research and Diagnostic Products) was used for quantitative measurement of human sRAGE levels, with a calibration range of 50–3200 pg/mL and a limit of detection at 19.2 pg/mL. Human esRAGE ELISA (Cusabio, CSB-E15773h) was used for quantitative measurement of human esRAGE. The calibration range for esRAGE was 0.625–40 ng/mL, with a limit of detection at 0.156 ng/mL. Human sRANKL (total) ELISA (Bio Vendor Research and Diagnostic Products) was used to establish sRANKL serum levels, with a calibration range of 31.25–2000 pg/mL and a limit of detection at 25 pg/mL. Human OPG ELISA (Bio Vendor Research and Diagnostic Products) was used for quantitative measurement of human OPG. The calibration range for OPG was 180–7200 pg/mL, with a limit of detection at 36 pg/mL. Coefficients of variation for the assays of OPG, sRANKL, sRAGE, and esRAGE are shown in Table 2.

**Table 2:** Coefficients of variation for assays of OPG, sRANKL, sRAGE, and esRAGE

Assay	Coefficient of variation	
	Intra-assay (%)	Inter-assay (%)
OPG	3.53	5.78
sRANKL	9.38	12.00
sRAGE	4.00	7.15
esRAGE	5.20	8.50

We also measured the white blood cell count, the percentage of neutrophils in venous blood, and plasma levels of C-reactive protein (CRP) and procalcitonin. In both groups, the cervical length was assessed with a vaginal probe placed in the vestibule of the vagina using ultrasound. The arithmetic mean of three subsequent measurements was used in the study. In every woman, a microbiological smear for aerobic bacteria culture was taken from the cervical canal during gynecological examination. In group A, after exclusion of diagnosis of intrauterine infection, we administered intravenous inflow of fenoterol at a dose ranging from 0.0035 to 0.005 mg/min as a tocolytic agent, until inhibition of uterine contractions. The pregnant women were also administered betamethasone in two 12-mg doses with a 24-h interval to accelerate fetal lung maturation.

Group A was categorized into subgroups by the duration of pregnancy from the diagnosis of threatened premature labor up to delivery, with a 7-day cut-off point. In group B, antibiotic agents were administered after diagnosis to extend the duration of pregnancy between rupture of the membranes and delivery. We administered 2 g of ampicillin and 300 mg of erythromycin every 6 h intravenously for 48 h. We subsequently administered 500 mg of amoxicillin every 8 h and 250 mg of erythromycin every 6 h for 5 days orally as a standard protocol. These women were also administered two 12-mg doses of betamethasone with a 24-h interval to accelerate fetal lung maturation, and we avoided administration of tocolytic agents. Group B was additionally divided into subgroups according to the duration of pregnancy from rupture of the membranes to delivery, with the cut-off point considered as 24 h.

### Statistical analysis

Statistical evaluation was performed using Statistica 10.0 PL software for Windows. The distribution of variables was checked using the non-parametric Shapiro–Wilk W test, and according to the results, values were further analyzed. The level of significance was set at p <0.05. For the presentation of non-normally distributed variables, the number of patients, range of values (minimum–maximum), median, and the first and third quartile values (Q1–Q3) were included in the descriptive statistics. The results for normally distributed variables are shown as the number of patients, arithmetical mean, and standard deviation (SD). The Mann–Whitney *U* test for unpaired variables was used to assess the differences in the studied parameters between the groups. For statistical analysis of relationships, correlation coefficients were estimated using Spearman’s test. Receiver operating characteristic (ROC) curve analysis was used to determine the cut-off point, as well as the predictive value of tests, their sensitivity, specificity, and positive and negative predictive values (PPV and NPV, respectively), and accuracy. Comparison of the area under the curve (AUC) was used to compare diagnostic tests.

## Results

The distribution of most values of the analyzed parameters was not normal (Shapiro–Wilk W-test; p > 0.05). Descriptive statistics of the variables are shown in Table 3. In group A, a positive correlation was found between sRAGE levels and the duration of pregnancy from the onset of symptoms of threatened premature labor until completion of delivery, and a negative correlation was found between sRANKL levels and the duration of pregnancy from diagnosis until delivery.

**Table 3:** Descriptive statistics of the study groups

Parameter	Group A					Group B				
	N	min–max	Q1	Q3	Median	N	min–max	Q1	Q3	Median
WBC (10^9^/L)	41	3.32–20.06	9.51	14.4	13.19	49	8.23–25.40	10.05	14.38	11.82
CRP (mg/L)	41	0.4–39.5	2.3	5.5	3.7	49	0.2–77.3	2.7	11.8	5.8
Band (%)	41	63.5–92.0	74.2	79.4	76.8	49	55.7–91.0	66.7	80.8	71.7
PCT (μg/L)	40	0.02–0.08	0.03	0.07	0.05	43	0.03–10.10	0.03	0.06	0.05
sRAGE (pg/mL)	41	128.7–1686.6	352.5	787.5	594.9	49	48.9–4872.0	297.2	775.3	612.9
esRAGE (pg/mL)	41	230.0–915.2	406.7	533.8	490.2	49	281.1–958.8	483.0	610.1	541.1
sRANKL (pg/mL)	41	2046.5–85,437.5	4374.4	9168.7	6425.0	49	1075.0–75,875.0	7250.0	29,381.2	11,362.5
OPG (pg/mL)	41	157.2–2048.4	332.3	1449.6	531.7	49	234.2–14,520	411.8	867.1	581.4

WBC: white blood cells; CRP: C-reactive protein; Band:banded neutrophils; PCT: procalcitonin; sRAGE:secretory receptors for advanced glycation end products; esRAGE:endogenous secretory receptors for advanced glycation end products; sRANKL:soluble receptor activator of nuclear factor κB; OPG:osteoprotegerin; Q1:quartile 1; Q3:quartile 3; min:minimum; max:maximum

**Fig. 1:**
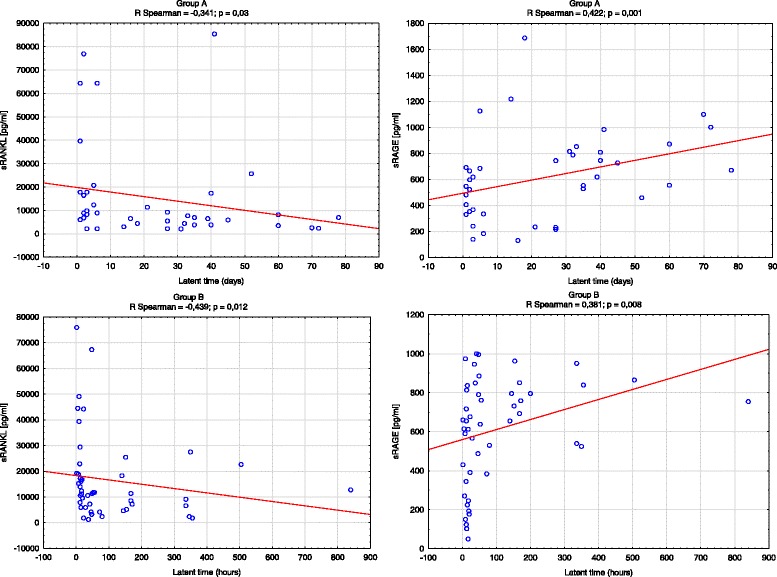
Two-dimensional scatterplots. The scatterplots show the correlation between sRAGE and sRANKL levels and the latent time from symptoms until delivery in both study groups

In group B, a positive correlation was found between sRAGE levels and the duration of pregnancy from pPROM until completion of delivery. There was also a negative correlation between sRANKL levels and the interval from pPROM until delivery (Fig. 1).

**Fig. 2:**
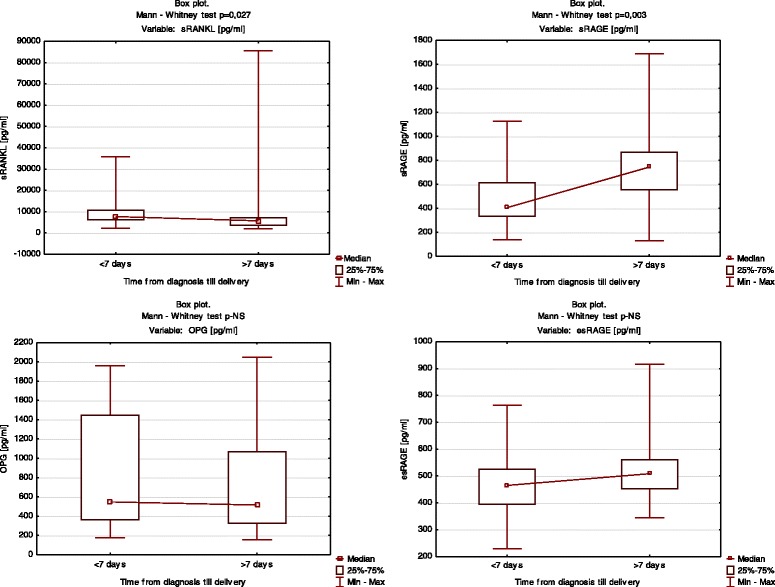
Box plots of Group A. Levels of sRAGE, esRAGE, OPG, and sRANKL according to latent time from symptoms until delivery. The Mann–Whitney *U*-test was used for comparison

In group A, a duration of pregnancy shorter than 7 days from diagnosis to delivery was accompanied by a lower sRAGE level and a higher sRANKL level (median = 405.9 pg/mL vs 744.0 pg/mL; median = 8253.1 pg/mL vs 5671.8 pg/mL, respectively, Fig. 2).

**Fig. 3:**
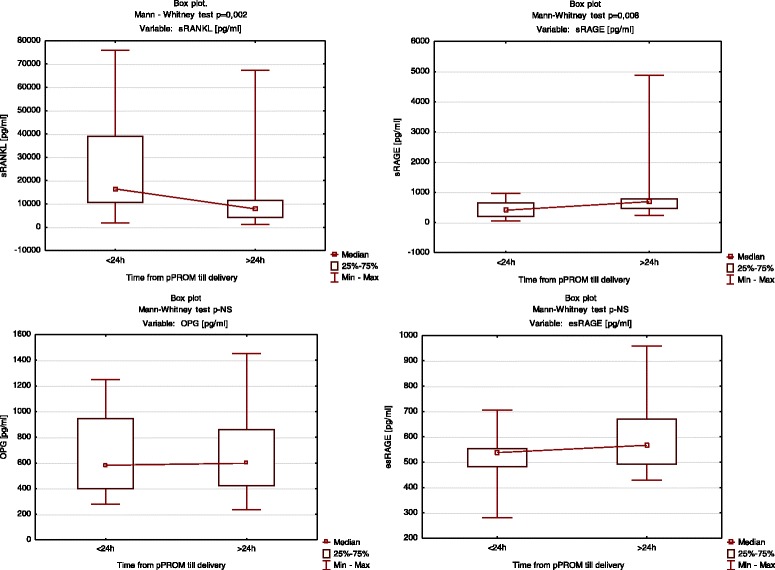
Box plots of Group B. Levels of sRAGE, esRAGE, OPG, and sRANKL according to latent time from symptoms until delivery, using the Mann–Whitney *U*-test

In group B, a duration of pregnancy shorter than 24 h from pPROM until delivery was accompanied by lower sRAGE levels and higher sRANKL levels (median = 410.6 pg/mL vs 712.05 pg/mL; median = 16,428.8 pg/mL vs 7868.7 pg/mL, respectively, Fig. 3).

**Fig. 4:**
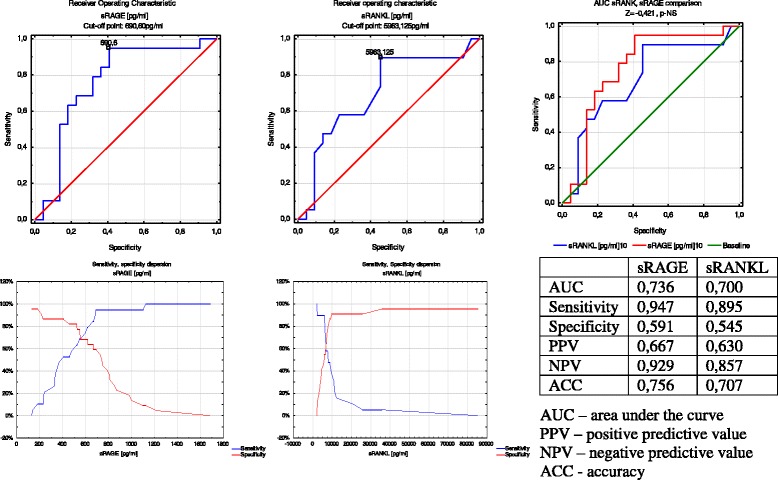
ROC curve analysis of sRAGEand sRANKL according to latent time from symptoms until delivery in group A. AUC: area under the curve; PPV: positive predictive value; NPV: negative predictive value; ACC:accuracy

In group A, analysis of the AUC showed a low risk of delivery in a 7-day period from diagnosis of threatened preterm labor for sRANKL levels lower than 5963.1 pg/mL. The sensitivity was 0.895 and the NPV was 0.857. Analysis of the AUC for sRAGE showed a low risk of premature delivery in a 7-day period from diagnosis of threatened preterm labor for sRAGE levels exceeding 690.6 pg/mL. The sensitivity was 0.947 and the NPV was 0.929. Comparison of the AUC for sRAGE and sRANKL showed similar prognostic values (Fig. 4).

**Fig. 5:**
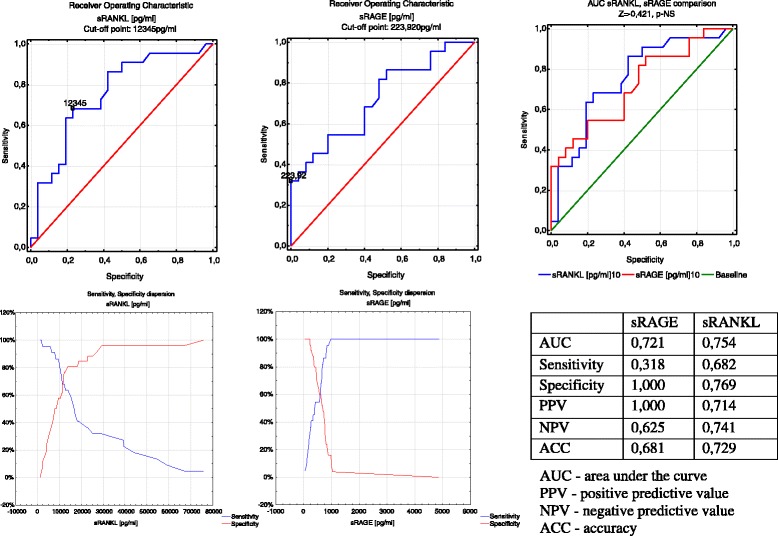
ROC curve analysis of sRAGE and sRANKL according to latent time from symptoms until delivery in group B. AUC: area under the curve; PPV: positive predictive value; NPV: negative predictive value; ACC: accuracy

In group B, analysis of the AUC for sRANKL showed that sRANKL levels lower than 12345.1 pg/mL predicted a low risk of preterm delivery in 24 h from pPROM. The sensitivity was 0.682 and the NPV was 0.741. Analysis of the AUC for sRAGE showed that when the sRAGE level was 223.92 pg/mL, the sensitivity was as low as 0.318, but the specificity and PPV reached 1.0. Comparison of the AUC for sRAGE and sRANKL showed a similar prognostic value (Fig. 5).

High sRANKL levels were correlated with positive results of a cervical microbiological smear (r = 0.383, p = 0.013). Comparison of the rank correlations in group A is shown in Table 4. In group B, high sRANKL levels were correlated with positive cervical microbiological smear findings (r = 0.356, p = 0.012) and low sRAGE levels. Comparison of rank correlations in group B is shown in Table 5.

**Table 4:** Correlations between serum sRAGE, esRAGE, sRANKL, and OPG levels and other markers in group A

Correlation	r	p	Correlation	r	p
esRAGE vs WBC	0.149	NS	sRANKL vs WBC	0.182	NS
esRAGE vs CRP	0.238	NS	sRANKL vs CRP	0.074	NS
esRAGE vs band	0.133	NS	sRANKL vs band	−0.094	NS
esRAGE vs MC	−0.165	NS	sRANKL vs MC	0.383	0.013
esRAGE vs PCT	0.368	NS	sRANKL vs PCT	0.051	NS
esRAGE vs GD	0.045	NS	sRANKL vs GD	−0.220	NS
esRAGE vs BW	0.038	NS	sRANKL vs BW	−0.071	NS
sRAGE vs WBC	0.070	NS	OPG vs WBC	−0.318	NS
sRAGE vs CRP	−0.303	NS	OPG vs CRP	0.383	0.048
sRAGE vs band	−0.171	NS	OPG vs band	0.280	NS
sRAGE vs MC	−0.165	NS	OPG vs MC	0.049	NS
sRAGE vs PCT	−0.453	NS	OPG vs PCT	0.448	NS
sRAGE vs GD	0.469	0.002	OPG vs GD	0.093	NS
sRAGE vs BW	0.338	0.03	OPG vs BW	0.072	NS

p: level of significance; r: Spearman’s correlation; sRAGE:secretory receptors for advanced glycation end products; esRAGE:endogenous secretory receptors for advanced glycation end products; sRANKL:soluble receptor activator of nuclear factor κB; OPG:osteoprotegerin; WBC:white blood cells; CRP: C-reactive protein; band: banded neutrophils; MC: microbial culture from the cervix; PCT:procalcitonin; GD: gestational age at delivery; BW: birth weight

**Table 5:** Correlations between serum sRAGE, esRAGE, sRANKL, and OPG levels and other markers in group B

Correlation	r	p	Correlation	r	p
esRAGE vs WBC	−0.030	NS	sRANKL vs WBC	0.113	NS
esRAGE vs CRP	0.390	0.020	sRANKL vs CRP	−0.072	NS
esRAGE vs band	0.035	NS	sRANKL vs band	0.218	NS
esRAGE vs MC	−0.174	NS	sRANKL vs MC	0.356	0.012
esRAGE vs PCT	−0.077	NS	sRANKL vs PCT	0.255	NS
esRAGE vs GD	0.069	NS	sRANKL vs GD	0.246	NS
esRAGE vs BW	0.038	NS	sRANKL vs BW	0.270	NS
sRAGE vs WBC	0.012	NS	OPG vs WBC	0.082	NS
sRAGE vs CRP	−0.293	NS	OPG vs CRP	0.164	NS
sRAGE vs band	−0.202	NS	OPG vs band	0.001	NS
sRAGE vs MC	−0.293	0.045	OPG vs MC	0.074	NS
sRAGE vs PCT	−0.099	NS	OPG vs PCT	0.301	NS
sRAGE vs GD	0.206	NS	OPG vs GD	−0.037	NS
sRAGE vs BW	0.078	NS	OPG vs BW	0.077	NS

p: level of significance; r: Spearman’s correlation; sRAGE:secretory receptors for advanced glycation end products; esRAGE:endogenous secretory receptors for advanced glycation end products; sRANKL:soluble receptor activator of nuclear factor κB; OPG: osteoprotegerin; WBC:white blood cells; CRP: C-reactive protein; band: banded neutrophils; MC: microbial culture from the cervix; PCT: procalcitonin; GD: gestational age at delivery; BW: birth weight

**Fig. 6:**
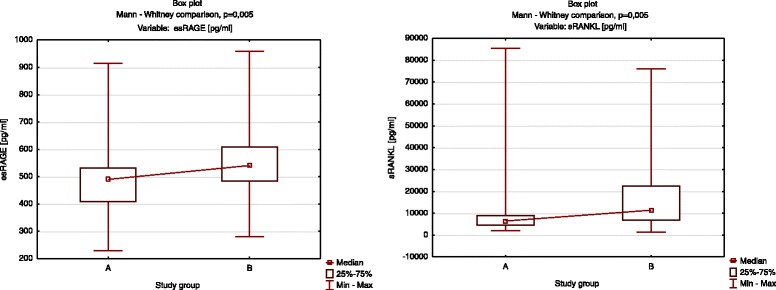
Comparison of esRAGE and sRANKL between the groups. The Mann–Whitney *U*-test was used for comparison between the groups

The median values of esRAGE and sRANKL levels were significantly lower in group A than in group B (median = 490.2 vs 541.1 pg/mL; 6425.0 vs 11362.5 pg/mL, respectively, Fig. 6). The values of the other variables were not significantly different between the groups (Table 6).

**Table 6:** Comparison ofstudy parameters between groups

Parameter	Rank-sum group A	Rank-sum group B	U	Z	p
WBC (10^9^/L)	1213.5	1487.5	652.50	−0.077	NS
CRP (mg/L)	1003.0	1625.0	475.00	−1.864	NS
Band (%)	623.5	504.5	228.50	1.000	NS
PCT (μg/L)	202.5	577.5	136.50	−0.530	NS
sRAGE (pg/mL)	1883.0	2122.0	946.00	0.308	NS
esRAGE (pg/mL)	1107.5	1973.5	477.50	−2.757	0.005
sRANKL (pg/mL)	1471.5	2623.5	610.50	−3.188	0.001
OPG (pg/mL)	1342.5	1660.5	714.50	0.164	NS

WBC:white blood cells; CRP: C-reactive protein; Band:banded neutrophils; PCT:procalcitonin; sRAGE:secretory receptors for advanced glycation end products; esRAGE:endogenous secretory receptors for advanced glycation end products; sRANKL:soluble receptor activator of nuclear factor κB; OPG:osteoprotegerin; U:Mann–Whitney *U* test; Z:Mann–Whitney Z test; p: Mann–Whitney level of significance

## Discussion

Many studies have focused on the role of the OPG/RANKL/RANK system, not only in osteoporosis, but also in cardiovascular and autoimmune (e.g., rheumatoid arthritis) diseases or neoplasms [43–49]. However, there is a lack of studies on assessment of components of the OPG/RANKL/RANK system in premature labor. Only a few studies have described the relationship between the OPG/RANKL/RANK system and pregnancy-induced hypertension, preeclampsia, and intrauterine growth restriction [50–55].

Negative RAGE isoforms can inhibit endogenous inflammation and their protective function has been confirmed in diabetes mellitus, some cardiovascular diseases, atherosclerosis, and in some types of neoplasms [56–62]. Only a few studies have assessed the importance of RAGE for preterm labor [39–42, 63]. These studies did not clearly prove a protective function of negative soluble RAGE isoforms in such complications of pregnancy. Additionally, only a few studies evaluated RAGE and sRANKL levels in threatened preterm labor. Romero et al. assessed RAGE levels in amniotic fluid in five groups of pregnant women: (1) women with a gestational age between 14 and 18 weeks of an uncomplicated pregnancy; (2) pregnancies at term; (3) women in labor at term; (4) pregnant women threatened with premature labor with unruptured fetal membranes; and (5) women diagnosed with pPROM, depending on the presence or absence of intrauterine infection [39]. The authors found that amniotic fluid sRAGE and esRAGE levels increased as pregnancy progressed, and they were positively correlated with intra-amniotic infection in preterm pregnancy. Considering the molecular patterns of RAGE function, the aforementioned findings are unexpected. However our finding of increased esRAGE levels in women who were diagnosed with pPROM is consistent with previous studies [57, 64, 65]. Another study showed decreased RAGE levels in women with overt chorioamnionitis [40]. This findingis consistent with the molecular theory of the biological function of RAGE.

These different previous findings led to our focus on analyzing biomarkers as risk factors for the outcome of preterm birth. We found a positive correlation between sRAGE levels and the interval from diagnosis to delivery in both groups. This finding suggests a protective function of RAGE. A protective role of increased sRAGE levels in threatened preterm labor was also found by Bastek et al. who analyzed plasma sRAGE levels in a large group of women (n = 529) with the threat of premature labor [42]. They found lower sRAGE levels in patients who gave birth prematurely compared with those who delivered at term. The authors concluded that evaluation of sRAGE may be a useful marker of premature labor, which is consistent with our findings. Germanova et al. showed decreased sRAGE levels in pregnant women suffering from threatened preterm delivery and from preeclampsia compared with healthy pregnant women, indicating a protective role of RAGE [66]. Both of the complications of pregnancy analyzed by Germanova et al. are characterized by chronic inflammation [67, 68]. Hajek et al. found lower sRAGE levels in women who were diagnosed with threatened preterm labor compared with those with healthy pregnancies [41]. They concluded that the presence of symptoms of threatened premature labor was associated with a decrease in RAGE levels. In the fetal membranes, expression of high-mobility group box-1,which is one of the RAGE ligands, is higher in preterm rupture of the membranes than at term, and promotes one of the mitogen-activated protein kinases (p38MAPK) associated with non-infectious inflammatory responses [69]. However, there are no conclusive data on negative RAGE isoform expression in preterm pregnancy. In our study, there was no association between gestational age and soluble RAGE levels. Based on the fact that premature aging is a reason of preterm delivery [70], a deficiency of the membrane-negative form of RAGE (dominant-negative RAGE) should be considered as a potential factor of aging of premature fetal tissue [63, 70, 71]. Our finding of a correlation between sRANKL and sRAGE levels and the latency period from diagnosis until delivery was the reason why we decided to evaluate the prognostic values of sRANKL and sRAGE for diagnosis of preterm labor for both study groups.

Seven days is an accepted cut-off point for the duration of the latency period in group A [72–74]. The sensitivity for sRANKL reached 89.5 % and the specificity was 54.4 %, with a PPV of 63 % and NPV of 85.7 %, while those for sRAGE were 94.7 %, 59.1 %, 66.7 %, and 92.9 %, respectively. Prognostic values of so-called classic risk markers of preterm labor are usually measured in symptomatic patients (i.e., fetal fibronectin and cervical length) and range from 60 % to 100 % [74–79]. Honest et al. analyzed the literature on prognostic values of tests that are used in calculating risk of preterm delivery, taking into account 319 published studies on evaluation of 22 tests [77]. The authors concluded that, despite the high sensitivity and specificity of investigated factors, such as the history of previous premature delivery, presence of fetal fibronectin in cervico-vaginal discharge, ultrasound cervical length measurements, and the level of some interleukins in amniotic fluid, their accuracy is still inadequate. This conclusionis supported by the fact that the prevalence of premature birth has not decreased.

In our study, the accuracy of sRAGE and sRANKL in group A was 75.6 % and 70.7 %, respectively. A high sensitivity and NPV, with a high accuracy, suggest that sRAGE and sRANKL could be new effective biomarkers of premature labor. The usefulness of detection of these markers in other compartments, such as amniotic fluid and cervical discharge, should also be assessed.

In group B, the 24-h cut-off point for the latency period was established. In most cases, pPROM is associated, as a cause or as a result, with intrauterine infection [80–83]. Spontaneous development of uterine contractility with subsequent completion of preterm delivery in 24 h from pPROM usually indicates rupture of membranes as a consequence of intrauterine infection. In such circumstances, prolongation of pregnancy, especially administration of tocolytic agents, could worsen the neonatal prognosis.

In our study, plasma sRAGE levels showed a low sensitivity (31.8 %), but a high specificity and PPV, both reaching 100 %. This finding suggests that when sRAGE levels lower than the cut-off point are found in a pregnant woman diagnosed with pPROM, completion of delivery in 24 h is practically guaranteed, although normal levels do not exclude the possibility of delivery. All of the prognostic sRANKL test values ranged between 70 % and 75 %. Thereis little information available to determine which pregnant women suffering from pPROM would deliver in a short period of time. Measurement of classic parameters of inflammation, such as CRP, white blood cells, interleukin-6, and others is useful, but not suitable as an ultimate predictor [7–9, 83].

In our study, we found higher sRANKL and esRAGE levels in pregnancies with the diagnosis of pPROM compared with those diagnosed with threatened preterm labor, but with intact membranes. Importantly, these differences could not have been caused by dissimilarity in gestational age because all valuations of parameters were made at a comparable stage of pregnancy (30.9 vs 31.1 weeks of gestation). We also excluded the influence of overt intrauterine infection because we obtained similar values of the white blood cell count, CRP levels, procalcitonin levels and band cell percentage, as well as the effect of glucocorticosteroids because betamethasone was administered to all of the patients. These results could have been due to an effect of increased activation of T-lymphocytes via cytokines in patients with pPROM compared with those with unruptured membranes. This suggests the presence of advanced inflammation in group B, but this was not confirmed by standard laboratory tests.

Analysis of the relationships between levels of sRANKL, OPG, sRAGE, and esRAGE with other parameters used in the diagnosis of premature labor showed a positive relationship between sRAGE levels and gestational age at delivery and birth weight in group A. This finding suggests a protective role of sRAGE in preterm pregnancy [41, 66].

Notably, in the entire studied population,we found an association between sRANKL levels and the results of cervical microbiological culture. A positive result of the culture was accompanied by elevated sRANKL levels preceding an increase in CRP levels or WBC. This finding may indicate sRANKL activation via pathogen-associated molecular patterns before other manifestations of infection.

The high predictive values for sRANKL and sRAGE that were obtained in both study groups indicate the potential of the usefulness of these markers in the diagnosis of preterm labor. However, our study has limitations, which include the small size of the study groups and theneed for validation.

## Conclusions

Correlations between sRANKL and sRAGE and the latent time from symptoms until delivery, as well as high prognostic values of sRANKL and sRAGE show the usefulness of these parameters in diagnosis of pregnant women with threatened premature labor. Further prospective research on larger study groups is required. A positive correlation between sRAGE levels and gestational age at delivery and birth weight in group A suggests the potential protective role of sRAGE in the pathogenesis of preterm labor. However, the relationship between sRANKL and cervical microbiological culture requires further study. Our finding of higher esRAGE and sRANKL levels in pregnant women suffering from pPROM compared with those whose fetal membranes remain intact suggests that they play a role in the pathogenesis of pPROM. Further research is required to determine the importance of sRANKL and esRAGE on the process of destruction of fetal membranes.

## Abbreviations

sRAGE: Secretory receptors for advanced glycation end products
esRAGE: Endogenous secretory receptors for advanced glycation end products
sRANKL: Soluble receptor activator of nuclear factor κB ligand
pPROM: Preterm premature rupture of the fetal membranes
OPG: Osteoprotegerin
TNF: Tumor necrosis factor
NF-κB: Nuclear factor kappa-B
RANK: Receptor activator of nuclear factor kappa-B
RAGE: Receptors for advanced glycation end products
MAPK: Mitogen-activated protein kinase
AGE: Advanced glycation end products
CRP: C-reactive protein
Q1–Q3: First and third quartile values
SD: Standard deviation
ROC: Receiver operating characteristic
PPV: Positive predictive value
NPV: Negative predictive value
AUC: Area under the ROC curve

## References

[1] Zou L, Wang X, Ruan Y, Li G, Chen Y, Zhang W. Preterm birth and neonatal mortality in China in 2011. Int J Gynaecol Obstet. 2014; http://dx.doi.org/10.1016/j.ijgo.2014.06.018. 10.1016/j.ijgo.2014.06.018 25132529

[2] Vitale SG, Marilli I, Rapisarda AM, Rossetti D, Belluomo G, Iapichino V (2014). Cellular and biochemical mechanisms, risk factors and management of preterm birth: state of the art. Minerva Ginecol.

[3] Passini R, Cecatti JG, Lajos GJ, Tedesco RP, Nomura ML, Dias TZ (2014). Brazilian Multicentre Study on Preterm Birth (EMIP): prevalence and factors associated with spontaneous preterm birth. PLoS ONE.

[4] Bastek JA, Sammel MD, Jackson TD, Ryan ME, McShea MA, Elovitz MA (2015). Environmental variables as potential modifiable risk factors of preterm birth in Philadelphia. Am J Obstet Gynecol.

[5] Grieger JA, Grzeskowiak LE, Clifton VL (2014). Preconception dietary patterns in human pregnancies are associated with preterm delivery. J Nutr.

[6] Vrachnis N, Vitoratos N, Iliodromiti Z, Sifakis S, Deligeoroglou E, Creatsas G (2010). Intrauterine inflamation and preterm delivery. Ann NY Acad Sci.

[7] Rzepka R, Torbé A, Czajka R, Kwiatkowski S, Bartoszek M, Cymbaluk A (2009). Rapid assessment of the IL-6 cervico-vaginal fluid level in threatening preterm labor. Ginekol Pol.

[8] Torbé A, Czajka R, Kordek A, Rzepka R, Kwiatkowski S, Rudnicki J (2007). Maternal serum proinflammatory cytokines in preterm labor with intact membranes: neonatal outcome and histological associations. Eur Cytokine Netw.

[9] Perales-Puchalt A, Brik M, Diago VJ, Perales A (2013). The negative predictive value of cervical interleukin-6 for the risk assessment of preterm birth. J Matern Fetal Neonatal Med.

[10] Timmons BC, Reese J, Socrate S, Ehinger N, Paria BC, Milne GL (2014). Prostaglandins are essential for cervical ripening in LPS-mediated preterm birth but not term or antiprogestin-driven preterm ripening. Endocrinology.

[11] Lockwood CL, Senyei AE, Dische M (1991). Fetal fibronectin in cervical and vaginal secretions as a predictor of preterm delivery. N Engl J Med.

[12] Lories RJ, Luyten FP (2001). Osteoprotegerin and osteoprotegerin-ligand balance: a new paradigm in bone metabolism providing new therapeutic targets. Clin Rheumatol.

[13] Martin TJ (2004). Paracrine regulation of osteoclast formation and activity: milestones in discovery. J Musculoskelet Neuronal Interact.

[14] Schoppet M, Preissner KT, Hofbauer LC (2002). RANK ligand and osteoprotegerin: paracrine regulators of bone metabolism and vascular function. Arterioscler Thromb Vasc Biol.

[15] Abedin M, Omland T, Ueland T, Khera A, Aukrust P, Murphy SA (2007). Relation of osteoprotegerin to coronary calcium and aortic plaque (from the Dallas Heart Study). Am J Cardiol.

[16] Olesen P, Ledet T, Rasmussen LM (2005). Arterial osteoprotegerin: increased amounts in diabetes and modifiable synthesis from vascular smooth muscle cells by insulin and TNF-alpha. Diabetologia.

[17] Rasmussen LM, Ledet T (2005). Osteoprotegerin and diabetic macroangiopathy. Horm Metab Res.

[18] Rogers A, Eastell R (2005). Circulating osteoprotegerin and receptor activator for nuclear factor kappaB ligand: clinical utility in metabolic bone disease assessment. J Clin Endocrinol Metab.

[19] Rogers A, Saleh G, Hannon RA, Greenfield D, Eastell R (2002). Circulating estradiol and osteoprotegerin as determinants of bone turnover and bone density in postmenopausal women. J Clin Endocrinol Metab.

[20] Lacey DL, Tan HL, Lu J, Kaufman S, Van G, Qiu W (2000). Osteoprotegerin ligand modulates murine osteoclast survival in vitro and in vivo. Am J Pathol.

[21] Ikeda T, Kasai M, Utsuyama M, Hirokawa K (2001). Determination of three isoforms of the receptor activator of nuclear factor-kappaB ligand and their differential expression in bone and thymus. Endocrinology.

[22] Suzuki J, Ikeda T, Kuroyama H, Seki S, Kasai M, Utsuyama M (2004). Regulation of osteoclastogenesis by three human RANKL isoforms expressed in NIH3T3 cells. Biochem Biophys Res Commun.

[23] Yasuda H, Shima N, Nakagawa N, Mochizuki SI, Yano K, Fujise N (1998). Identify of osteoclastogenesis inhibitory factor (OCIF) and osteoprotegerin (OPG): a mechanism by which OPG/OCIF inhibits osteoclastogenesis in vitro. Endocrinology.

[24] Hofbauer LC, Lacey DL, Dunstan CR, Spelsberg TC, Riggs BL, Khosla S (1999). Interleukin-1beta and tumor necrosis factor-alpha, but not interleukin-6, stimulate osteoprotegerin ligand gene expression in human osteoblastic cells. Bone.

[25] Takai H, Kanematsu M, Yano K, Tsuda E, Higashio K, Ikeda K (1998). Transforming growth factor-beta stimulates the production of osteoprotegerin/osteoclastogenesis inhibitory factor by bone marrow stromal cells. J Biol Chem.

[26] Walsh MC, Choi Y (2014). Biology of the RANKL-RANK-OPG system in immunity, bone, and beyond. Front Immunol.

[27] Anderson DM, Maraskovsky E, Billingsley WL, Dougall WC, Tometsko ME, Roux ER (1997). A homologue of the TNF receptor and its ligand enhance T-cell growth and dendritic-cell function. Nature.

[28] Iwamoto K, Miyamoto T, Sawatani Y, Hosogane N, Hamaguchi I, Takami M (2004). Dimer formation of receptor activator of nuclear factor kappaB induces incomplete osteoclast formation. Biochem Biophys Res Commun.

[29] Aubin JE, Bonnelye E (2000). Osteoprotegerin and its ligand: A new paradigm for regulation of osteoclastogenesis and bone resorption. Medscape Womens Health.

[30] Fu Q, Jilka RL, Manolagas SC, O’Brien CA (2002). Parathyroid hormone stimulates receptor activator of NFkappa B ligand and inhibits osteoprotegerin expression via protein kinase A activation of cAMP-response element-binding protein. J Biol Chem.

[31] Ramasamy R, Shi Fang Y, Herold K, Clynes R, Schmidt AM (2008). Receptor for advanced glycation end products. Fundamental roles in the inflammatory response: winding the way to the pathogenesis of endothelial dysfunction and atherosclerosis. Ann NY Acad Sci.

[32] Basta G, Lazzerini G, Massaro M, Simoncini T, Tanganelli P, Fu C (2002). Advanced glycation end products activate endothelium through signal-transduction receptor RAGE: a mechanism for amplification of inflammatory responses. Circulation.

[33] Ding Q, Keller JN (2005). Evaluation of rage isoforms, ligands, and signaling in the brain. Biochim Biophys Acta.

[34] Hanford LE, Enghild JJ, Valnickova Z, Petersen SV, Schaefer LM, Schaefer TM (2004). Purification and characterization of mouse soluble receptor for advanced glycation end products (sRAGE). J Biol Chem.

[35] Yonekura H, Yamamoto Y, Sakurai S, Petrova RG, Abedin MJ, Li H (2003). Novel splice variants of the receptor for advanced glycation end-products expressed in human vascular endothelial cells and pericytes, and their putative roles in diabetes-induced vascular injury. Biochem J.

[36] Romero R, Miranda J, Chaiworapongsa T, Korzeniewski SJ, Chaemsaithong P, Gotsch F (2014). Prevalence and clinical significance of sterile intra-amniotic inflammation in patients with preterm labor and intact membranes. Am J Reprod Immunol.

[37] Dubicke A, Andersson P, Fransson E, Andersson E, Sioutas A, Malmström A (2010). High-mobility group box protein 1 and its signalling receptors in human preterm and term cervix. J Reprod Immunol.

[38] Buhimschi CS, Baumbusch MA, Dulay AT, Oliver EA, Lee S, Zhao G (2009). Characterization of RAGE, HMGB1, and S100beta in inflammation-induced preterm birth and fetal tissue injury. Am J Pathol.

[39] Romero R, Espinoza J, Hassan S, Gotsch F, Kusanovic JP, Avila C (2008). Soluble receptor for advanced glycation end products (sRAGE) and endogenous secretory RAGE (esRAGE) in amniotic fluid: modulation by infection and inflammation. J Perinat Med.

[40] Romero R, Chaiworapongsa T, Savasan ZA, Hussein Y, Dong Z, Kusanovic JP (2012). Clinical chorioamnionitis is characterized by changes in the expression of the alarmin HMGB1 and one of its receptors, sRAGE. J Matern Fetal Neonatal Med.

[41] Hájek Z, Germanová A, Koucký M, Zima T, Kopecký P, Vítkova M (2008). Detection of feto-maternal infection/inflammation by the soluble receptor for advanced glycation end products (sRAGE): results of a pilot study. J Perinat Med.

[42] Bastek JA, Brown AG, Foreman MN, McShea MA, Anglim LM, Adamczak JE (2012). The soluble receptor for advanced glycation end products can prospectively identify patients at greatest risk for preterm birth. J Matern Fetal Neonatal Med.

[43] Knevel R, de Rooy DP, Saxne T, Lindqvist E, Leijsma MK, Daha NA (2014). A genetic variant in osteoprotegerin is associated with progression of joint destruction in rheumatoid arthritis. Arthritis Res Ther.

[44] Geusens P (2012). The role of RANK ligand/osteoprotegerin in rheumatoid arthritis. Ther Adv Musculoskelet Dis.

[45] Xu S, Wang Y, Lu J, Xu J (2012). Osteoprotegerin and RANKL in the pathogenesis of rheumatoid arthritis-induced osteoporosis. Rheumatol Int.

[46] Xi L, Cao H, Chen Y (2013). OPG/RANK/RANKL axis in atrial fibrillation. Cardiology.

[47] Irtiuga OB, Zhiduleva EV, Dubrovskaia OB, Moiseeva OM (2014). Concentration of osteoprotegerin and RANKL in blood serum of patients with aortic stenosis. Kardiologiia.

[48] Mori K, Le Goff B, Berreur M, Riet A, Moreau A, Blanchard F (2007). Human osteosarcoma cells express functional receptor activator of nuclear factor-kappa B. J Pathol.

[49] Mori K, Berreur M, Blanchard F, Chevalier C, Guisle-Marsollier I, Masson M (2007). Receptor activator of nuclear factor-kappaB ligand (RANKL) directly modulates the gene expression profile of RANK-positive Saos-2 human osteosarcoma cells. Oncol Rep.

[50] Briana DD, Boutsikou M, Boutsikou T, Malamitsi-Puchner A (2013). Relationships between maternal novel adipocytokines and bone biomarkers in complicated by gestational hypertensive disorders and normal pregnancies. J Matern Fetal Neonatal Med.

[51] Shen P, Gong Y, Wang T, Chen Y, Jia J, Ni S (2012). Expression of osteoprotegerin in placenta and its association with preeclampsia. PLoS ONE.

[52] Vitoratos N, Lambrinoudaki I, Rizos D, Armeni E, Alexandrou A, Creatsas G (2011). Maternal circulating osteoprotegerin and soluble RANKL in pre-eclamptic women. Eur J Obstet Gynecol Reprod Biol.

[53] Dorota DK, Bogdan KG, Mieczyslaw G, Bozena LG, Jan O (2012). The concentrations of markers of bone turnover in normal pregnancy and preeclampsia. Hypertens Pregnancy.

[54] Briana DD, Boutsikou M, Baka S, Hassiakos D, Gourgiotis D, Malamitsi-Puchner A (2009). Circulating osteoprotegerin and sRANKL concentrations in the perinatal period at term. The impact of intrauterine growth restriction. Neonatology.

[55] Madarász E, Tamás G, Tabák AG, Speer G, Lakatos P, Kerényi Z (2009). Osteoprotegerin levels in women with prior gestational diabetes mellitus. Diabetes Care.

[56] Schmidt AM, Hori O, Chen JX, Li JF, Crandall J, Zhang J (1995). Advanced glycation end products interacting with their endothelial receptor induce expression of vascular cell adhesion molecule-1 (VCAM-1) in cultured human endothelial cells and in mice. A potential mechanism for accelerated vasculopathy of diabetes. J Clin Invest.

[57] Fujisawa K, Katakami N, Kaneto H, Naka T, Takahara M, Sakamoto F (2013). Circulating soluble RAGE as a predictive biomarker of cardiovascular event risk in patients with type 2 diabetes. Atherosclerosis.

[58] Grossin N, Wautier MP, Meas T, Guillausseau PJ, Massin P, Wautier JL (2008). Severity of diabetic microvascular complications is associated with a low soluble RAGE level. Diabetes Metab.

[59] Daffu G, del Pozo CH, O’Shea KM, Ananthakrishnan R, Ramasamy R, Schmidt AM (2013). Radical roles for RAGE in the pathogenesis of oxidative stress in cardiovascular diseases and beyond. Int J Mol Sci.

[60] Colhoun HM, Betteridge DJ, Durrington P, Hitman G, Neil A, Livingstone S (2011). Total soluble and endogenous secretory receptor for advanced glycation end products as predictive biomarkers of coronary heart disease risk in patients with type 2 diabetes: an analysis from the CARDS trial. Diabetes.

[61] Piarulli F, Lapolla A, Ragazzi E, Susana A, Sechi A, Nollino L (2013). Role of endogenous secretory RAGE (esRAGE) in defending against plaque formation induced by oxidative stress in type 2 diabetic patients. Atherosclerosis.

[62] Moy KA, Jiao L, Freedman ND, Weinstein SJ, Sinha R, Virtamo J (2013). Soluble receptor for advanced glycation end products and risk of liver cancer. Hepatology.

[63] Rzepka R, Dołęgowska B, Rajewska A, Kwiatkowski S. On the significance of new biochemical markers for the diagnosis of premature labour. Mediators of Inflammation. 2014; doi:10.1155/2014/251451. 10.1155/2014/251451 PMC4274839 25548433

[64] Bierhaus A, Stern DM, Nawroth PP (2006). RAGE in inflammation: a new therapeutic target?. Curr Opin Investig Drugs.

[65] Wautier MP, Chappey O, Corda S, Stern DM, Schmidt AM, Wautier JL (2001). Activation of NADPH oxidase by AGE links oxidant stress to altered gene expression via RAGE. Am J Physiol Endocrinol Metab.

[66] Germanová A, Muravská A, Jáchymová M, Hájek Z, Koucký M, Mestek O (2012). Receptor for advanced glycation end products (RAGE) and glyoxalase I gene polymorphisms in pathological pregnancy. Clin Biochem.

[67] Laresgoiti-Servitje E (2013). A leading role for the immune system in the pathophysiology of preeclampsia. J Leukoc Biol.

[68] Romero R, Dey SK, Fisher SJ. Preterm labor: one syndrome, many causes. Science. 2014; http://dx.doi.org/10.1126/science.1251816. 10.1126/science.1251816 PMC4191866 25124429

[69] Bredeson S, Papaconstantinou J, Deford JH, Kechichian T, Syed TA, Saade GR (2014). HMGB1 promotes a p38MAPK associated non-infectious inflammatory response pathway in human fetal membranes. PLoS ONE.

[70] Menon R (2014). Oxidative stress damage as a detrimental factor in preterm birth pathology. Front Immunol.

[71] Menon R, Boldogh I, Hawkins HK, Woodson M, Polettini J, Syed TA (2014). Histological evidence of oxidative stress and premature senescence in preterm premature rupture of the human fetal membranes recapitulated in vitro. Am J Pathol.

[72] Liong S, Di Quinzio M, Fleming G, Permezel M, Rice G, Georgiou H (2015). New biomarkers for the prediction of spontaneous preterm labour in symptomatic pregnant women: a comparison with fetal fibronectin. BJOG.

[73] van Baaren GJ, Vis JY, Wilms FF, Oudijk MA, Kwee A, Porath MM (2014). Predictive value of cervical length measurement and fibronectin testing in threatened preterm labor. Obstet Gynecol.

[74] Melamed N, Hiersch L, Domniz N, Maresky A, Bardin R, Yogev Y (2013). Predictive value of cervical length in women with threatened preterm labor. Obstet Gynecol.

[75] Anwar A, Lindow SW, Greaves L, Hall S, Jha R (2014). The use of fetal fibronectin in suspected pre-term labour. J Obstet Gynaecol.

[76] Boots AB, Sanchez-Ramos L, Bowers DM, Kaunitz AM, Zamora J, Schlattmann P (2014). The short-term prediction of preterm birth: a systematic review and diagnostic metaanalysis. Am J Obstet Gynecol.

[77] Honest H, Hyde CJ, Khan KS (2012). Prediction of spontaneous preterm birth: no good test for predicting a spontaneous preterm birth. Curr Opin Obstet Gynecol.

[78] Abbott DS, Radford SK, Seed PT, Tribe RM, Shennan AH (2013). Evaluation of a quantitative fetal fibronectin test for spontaneous preterm birth in symptomatic women. Am J Obstet Gynecol.

[79] Di Renzo GC, Giardina I, Coata G, Di Tommaso M, Facchinetti F, Petraglia F (2011). Identification of preterm labor: the role of the fibronectin and ultrasound cervicometry and their association. Minerva Ginecol.

[80] Blencowe H, Cousens S, Oestergaard M, Chou D, Moller AB, Narwal R (2012). National, regional and worldwide estimates of preterm birth. Lancet.

[81] Lannon SM, Vanderhoeven JP, Eschenbach DA, Gravett MG, Adams Waldorf KM (2014). Synergy and interactions among biological pathways leading to preterm premature rupture of membranes. Reprod Sci.

[82] Dagklis T, Petousis S, Margioula-Siarkou C, Mavromatidis G, Kalogiannidis I, Prapas N (2013). Parameters affecting latency period in PPROM cases: a 10-year experience of a single institution. J Matern Fetal Neonatal Med.

[83] Simhan HN, Canavan TP (2005). Preterm premature rupture of membranes: diagnosis, evaluation and management strategies. BJOG.

